# *‘What the herbal medicine can do for me in a week, the orthodox does in a year’:* Perceived efficacy of local alternative therapies influences medication adherence in patients with atherosclerotic cardiovascular disease

**DOI:** 10.1111/hex.13185

**Published:** 2021-02-02

**Authors:** Amos Laar, Ernest Amoah Ampah, Yolanda Fernandez, Gideon Senyo Amevinya, Priscillia Nortey, Frank Benyah, Joseph Akamah, Marcella Ambenne, Peter Lamptey, Caroline Free, Helena Legido‐Quigley, Pablo Perel

**Affiliations:** ^1^ Department of Population, Family, & Reproductive Health School of Public Health University of Ghana, Legon Accra Ghana; ^2^ House of Mentoring and Research Resources (HM2R). Box LG 1099 University Post Office, Legon Accra Ghana; ^3^ Department of Non‐communicable Disease Epidemiology Faculty of Epidemiology and Population Health London School of Hygiene & Tropical Medicine. Keppel St London UK; ^4^ Department of Epidemiology and Disease Control School of Public Health Box LG 13 University of Ghana, Legon Accra Ghana; ^5^ The Ghana Police Hospital Accra Ghana; ^6^ Department of Medicine and Therapeutics Divisions of Cardiology and Neurology School of Medicine and Dentistry University of Ghana Korle‐Bu Ghana; ^7^ Department of Population Health Faculty of Epidemiology and Population Health London School of Hygiene & Tropical Medicine Keppel St London UK

**Keywords:** atherosclerotic cardiovascular disease, faith‐based healing, Ghana, herbal therapies, Non‐adherence, orthodox medication, qualitative research

## Abstract

**Background:**

There is strong evidence that anti‐platelet therapy, ACE inhibitors, beta‐blockers and statins are cost‐effective in reducing subsequent cardiovascular disease (CVD) events in patients with atherosclerotic cardiovascular disease (ACVD). In some settings, only a low proportion of people have access to these medications, and even lower adhere to them. The current study explored and presents data on the causes of poor adherence to orthodox medication and motivations for alternative therapies in patients with established atherosclerotic cardiovascular disease (ACVD).

**Methods:**

The study was conducted among city‐dwelling adults with ACVD in Accra – Ghana's capital city. Eighteen interviews were conducted with patients with established ACVD. A follow‐up focus group discussion was conducted with some of them. The protocol was approved by two ethics review committees based in Ghana and in the United Kingdom. All participants were interviewed after informed consent. Analysis was done with the Nvivo qualitative data analysis software.

**Results:**

We identified motivations for use of alternatives to orthodox therapies. These cover the five dimensions of adherence: social and economic, health‐care system, condition‐related, therapy‐related, and patient‐related dimensions. Perceived inability of an orthodox medication to provide immediate benefit is an important motivator for use of alternative forms of medication.

**Conclusions:**

A multiplicity of factors precipitate non‐adherence to orthodox therapies. Perceived efficacy and easy access to local alternative therapies such as herbal and faith‐based therapies are important motivators.

## 1 INTRODUCTION

Every year, over 30 million people experience acute coronary event or stroke; one‐quarter of these events occur in people with established atherosclerotic cardiovascular disease (ACVD).^1^ ACVD includes two major conditions: ischaemic heart disease (IHD) and cerebrovascular disease (mainly ischaemic stroke). These conditions accounted for a combined 15.2 million deaths worldwide in the year 2016 and are the first and second causes of death worldwide.^2^ By 2030, more than 23.3 million people are expected to die annually from cardiovascular diseases (CVDs).^2^

According to the World Health Organization (WHO), at least 75% of deaths due to CVDs occur in low‐ and middle‐income countries (LMICs).^3^ In these settings, the arterial events referred to above occur at an earlier age, affecting economically active populations and have a large economic impact.^4^ For instance, Agyemang et al showed that more than 50% of CVD‐related deaths in Africa occur among persons 30‐69 years of age, which is 10 years or more below the equivalent group in the developed world.^5^ There are even bleaker projections that Africa's CVD burden will continue to rise and by 2020 it will double the burden in 1990.^6^

In Ghana, the WHO identified CVD as one of the top two causes of death after diarrhoeal disease.^7^ The probability of dying from CVD in Ghana for individuals between the ages 30 and 70 is 20%.^8^ Trial evidence elsewhere has shown that in patients with established ACVD, medicines such as anti‐platelet therapy, ACE inhibitors, beta‐blockers and statins are cost‐effective in reducing the risk of subsequent cardiovascular disease (CVD) events. Furthermore, the use of these medicines reduces the need for hospitalization, increase longevity and improve the health‐related quality of life of the individual.^9^, ^10^, ^11^, ^12^ As a result, they are recommended in clinical guidelines and included in the WHO Essential Medicines List (EML).^13^

Unfortunately, not all those who are in need of these medications take them. The PURE study, for instance, showed that in low‐income countries only 1 in 5 patients with ACVD are receiving this group of cardiovascular drugs.^14^ Among those with access to these medications, suboptimal adherence is a major problem. There is abundant evidence that in LMICs adherence to these cardiovascular medications in patients with ACVD is poor. Kronish and Ye (2013) have shown that approximately 50% of patients with cardiovascular disease or its major risk factors have poor adherence to their prescribed medication.^15^

Many factors have been linked to non‐adherence to prescribed orthodox medications by patients with ACVDs. First, health expectations in the management and survival of ACVD patients are directly linked to the adherence to the conventional treatment regimen. There are other reasons – including patients’ stress and psychological state, the availability, accessibility, affordability and perceived efficacy of alternatives which may affect adherence.^16^, ^17^, ^18^ Patients’ perceptions of the disease (eg fatalistic perceptions, absence of symptoms) and the medications (eg fear of side‐effects, confusion about multiple medications); patient–physician relationships; availability of family/social network support; and co‐morbidities (eg depression)^19^, ^20^, ^21^ are other important factors. Available literature shows the tendency to focus on patient‐related factors as the cause of medication non‐adherence, to the relative neglect of factors such as those related to the health system. For this reason, the WHO regards adherence as a multidimensional phenomenon determined by the interplay of at least five sets of factors, or ‘dimensions’ of which patient‐related factors are just one determinant.^22^ We describe below some of these determinants/inter‐related factors.

### 1.1 Therapy‐related factors

There are many therapy‐related factors that affect adherence. Most notable are those related to the complexity of the medical regimen, poly‐pharmacy and the adverse reactions engendered, previous treatment failures, frequent changes in treatment, the immediacy of beneficial effects, side‐effects^23^, ^24^, ^25^, ^26^, ^27^ and the availability of medical support to deal with them.

### 1.2 Health‐care system‐related factors

Effective communication requires providing medication instructions at a level that the patient can understand. The ability of health‐care providers to spend time communicating appropriately with a patient, their communication and interpersonal style have been found to be important in determining the level of adherence to medication.^28^, ^29^ Several studies have shown that inadequate information and medical care coupled with poorly developed health services, non‐existent health insurance plans and low doctor‐to‐patient ratio all impact negatively on medication adherence.^22^, ^30^ In a qualitative study exploring the barriers to medication adherence among patients with uncontrolled diabetes, some patients indicated that seeing more than one care provider for the same issue could create confusion and complications in the care management plan.^31^

### 1.3 Social and economic factors

Some factors reported to have a significant effect on adherence are poverty, illiteracy, low level of education, gender roles, unemployment, lack of effective social support networks, unstable living conditions, and long distance from treatment centre.^22^, ^30^, ^32^, ^33^ Health literacy is an acknowledged determinant of medication adherence.^34^, ^35^ Furthermore, societal norms in Ghana which breed suspicion about science‐based medication have been found to negatively affect the adherence to medication.^25^

### 1.4 Condition‐related factors

These factors refer to the demands the disease condition places on the patient. These may include severity of symptoms, severity of disability, and severity of disease.^22^ These influence patients’ risk perception and determine the level of importance the patient will attach to adhering to their medication regimen. It has been found for example that patients rated in poor health by their physicians were more likely to adhere to their medication.^36^

### 1.5 Herbal and faith‐based healing

For those in Ghana and similar African settings, high dependence on herbal and other non‐orthodox alternatives is an important reason for non‐adherence to prescribed therapies.^18^, ^37^, ^38^ The vast majority of the African people (80%), according to the WHO, rely on traditional medicine for their primary health‐care need.^39^ Mander et al^39^ note that many Africans do not consider traditional medicine an inferior alternative to western medicine. To them, traditional medicine is desirable and necessary for treating a range of health problems that western medicine does not treat adequately.^39^ The Ghana Herbal Pharmacopoea (GHP) reveals that about 70% of Ghanaians depend on alternative health‐care practices for their primary health‐care needs.^40^ Several reports note that the use of herbal preparation for the management of ailments including ACVD is widespread in Ghana due to their perceived accessibility, affordability and perceived efficacy as compared to orthodox medicine.^41^, ^42^, ^43^, ^44^ Others cite the inadequate number of health facilities coupled with the limited availability of trained personnel.^45^, ^46^ There are also reports that herbal medicine use is on the ascendency due to pressure from peers, family and relatives.^25^

In Ghana and other African countries, there is the notion that certain diseases are as a result of curses or angry deities and spells created by witchcraft. Certain illnesses are also seen as spiritual when they persist even after the patient has received treatment from a medical doctor or a herbalist.^47^ To cure these diseases therefore, there is the need to pray for God's forgiveness. Faith‐based healers make claims of accessing the divine and providing a cure for various chronic ailments. The belief by some patients that spiritual healers have the power to cure disease conditions influences adherence to these medications. In a study to determine the consequence of religious beliefs on medication adherence among hypertensive patients in Ghana, it was observed that a lower likelihood of adhering to treatment was associated with high spiritual beliefs of patients.^42^

Given the high prevalence of people with established ACVD in LMICs and that improvement in adherence to cardiovascular medications has been associated with improved survival (~15%‐19% relative risk reduction of CVD events), any effort aimed at improving medication adherence has a great potential to improve population health.^48^ The purpose of this paper is to better understand how patients with established atherosclerotic cardiovascular disease (ACVD) in one middle income country, Ghana, adhere (or do not adhere) to necessary medication and causes of poor adherence to cardiovascular secondary prevention medications with a particular focus on the perceived efficacy of local alternative therapies.

## 2 METHODS

### 2.1 Study design

**FIGURE 1:**
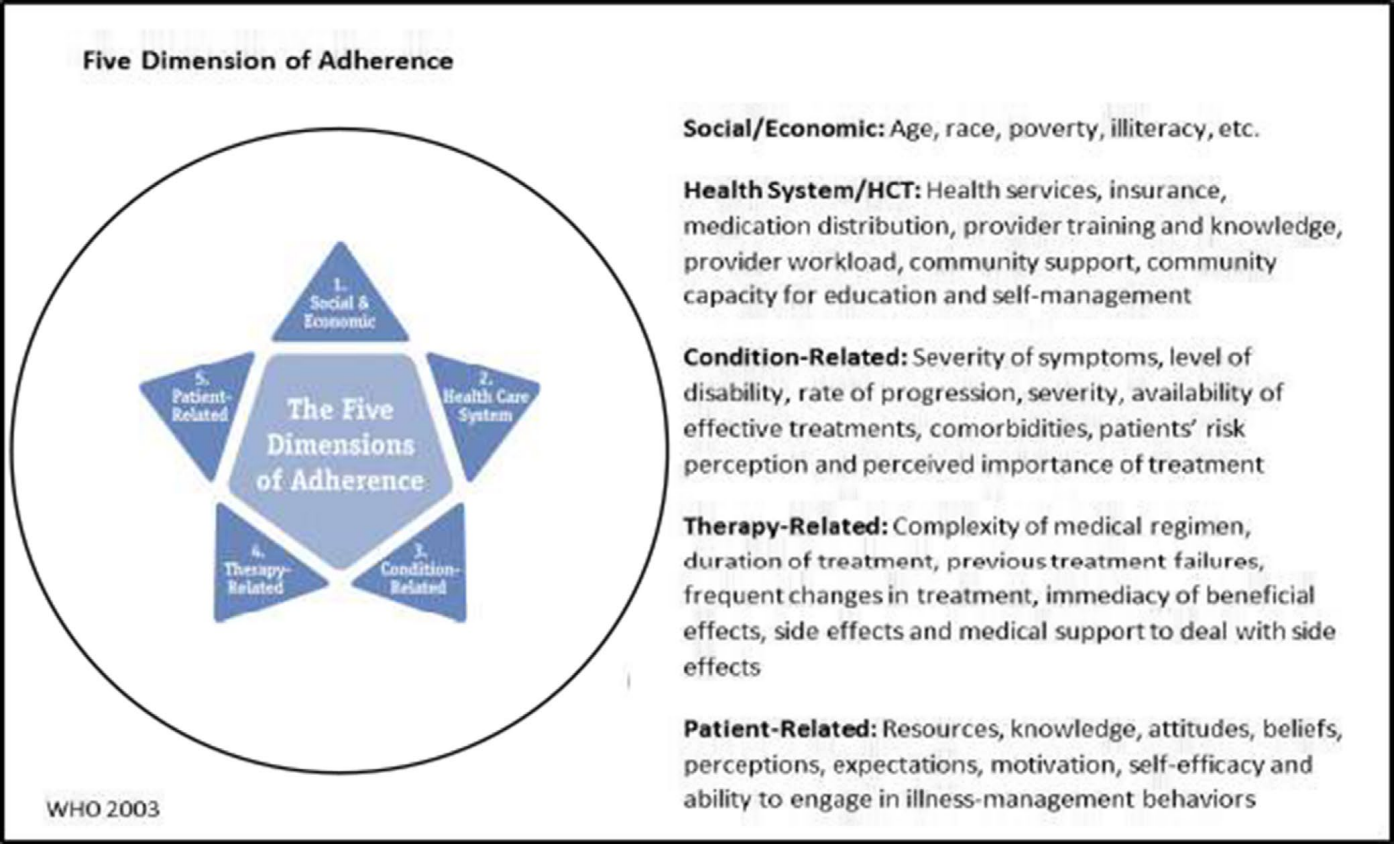
Five dimensions of adherence

To explore factors associated with medication adherence in patients with established ACVD, a qualitative study design, deploying semi‐structured interview and focus group discussions was conducted. This design was part of a larger study ( Global Txt2heart Pilot Study) which deployed a sequential mixed‐methods design – a qualitative phase, followed with a quantitative phase. The qualitative phase identified key factors leading to poor adherence to CVD medications used for secondary prevention among the target population. The design, analysis and presentation of the data drive motivations from the WHO’s Five Interacting Dimensions of Adherence (see Figure 1).^22^

### 2.2 Study settings

This study was conducted in the Greater Accra Region of Ghana among patients diagnosed of ACVD and receiving treatment at the Police Hospital, Accra. The Police Hospital provides health‐care services for members of the Ghana Police Service and their families as well as the general public, mostly patients within the national capital, Accra. It also serves as a referral point to and from other health facilities. The pilot study was designed to be implemented in a typical health facility in Ghana's national capital – to generate data that facilitate scale‐up in other facilities in the setting. Given resource constraints and other considerations (eg capacity to implement and test the planned mHealth intervention), the profile of the Police Hospital matched our definition of a typical health facility. Further consideration to our choice was the fact that the scale‐up phase of the intervention will involve government, quasi‐government and potential private facilities.

### 2.3 Study respondents

Respondents were adults (>18 years old) of both genders, who owned a mobile phone and had at least 6 months of history of established ACVD defined as coronary artery disease (including non‐primary coronary revascularizations), ischaemic stroke, peripheral artery disease and atherosclerotic aortic disease in whom anti‐platelet, BP lowering medications and statins were recruited to partake in the study. Aware that, the intervention phase of the study would require ownership or access to a mobile phone – a platform via which patients/participants receive medication adherence intervention in the form of short messages services (SMS), ownership of a mobile phone served as one of the inclusion criteria. A maximum variation sample of 18 respondents selected (with consideration given to gender, age, location, medication adherence status and socio‐economic status) participated in the semi‐structured interviews. Six of the 18, who were purposively selected, agreed to participate in the focus group discussion. There was no prior relationship between the study participants and the researchers (except co‐author FB; a primary care physician for our study participants).

### 2.4 Data collection

Two qualitative data collection methods – face‐to‐face semi‐structured interviews and focus group discussions – were conducted by two experienced multilingual qualitative field researchers fluent in English and the two most commonly spoken Ghanaian languages in the study setting – Ga and Twi. Eighteen interviews were scheduled when ACVD patients visited the health facility for care. The duration for these interviews was between thirty and fifty minutes. A follow‐up focus group discussion was conducted within the premises of the health facility. The focus group discussion lasted about an hour and thirty minutes. Data collection was preceded by obtaining consent from the medical facility to provide files on patients with ACVD diagnoses.

### 2.5 Data analysis, reporting and quality assurance

Captured audio recordings of interviews and focus groups were translated directly to English (where applicable) and transcribed verbatim by experts fluent in Ga or Twi and English languages. Themes were initially developed and transcripts and notes from the interviews were coded using Nvivo software (Version 11). Transcripts were coded by a data coder, adopting a mixed inductive and deductive approach using techniques drawn from grounded theory such as line by line analysis and the use of the constant comparative method. Co‐authors AL, YF, EAA and GSA met to discuss major themes and issues that were not clear in the transcriptions. Data reporting was done according to the consolidated criteria for reporting qualitative research (COREQ).^49^

### 2.6 Ethics considerations

Our study protocol obtained ethical approval from the Ghana Health Service Ethics Review Committee (Ref # GHS‐ERC:06‐09‐2016) and the London School of Hygiene and Tropical Medicine (Ref. # 12046). All interviews were conducted after informed consent.

## 3 RESULTS

Our findings are organized around five main themes identified from the WHO’s Five Interacting Dimensions of Adherence.

### 3.1 Patient‐related dimension

Patient‐related dimension of adherence refers to the resources, attitudes and the perception the patient has about the treatment regimen. The analysis identified ‘forgetfulness’ and ‘the availability of resources’ emerged as sub‐themes.

#### 3.1.1 Forgetfulness

Some patients reported that they forgot to take their medication thus negatively impacting adherence. The following quotes describe examples of how patients forgot to take their medication because of tiredness or pre‐occupation with chores:

‘Yes, at times when I’m busy doing some other chores, I do forget. Sometimes when I’m supposed to take the medication in the morning but I haven't eaten early I do forget to take...’ [IDI, female, 67 years].

#### 3.1.2 Resources

Some study participants reported that their inability to purchase their medication resulted in non‐adherence. Below are quotes that describe how the lack of resources led to non‐adherence which in some cases resulted in the worsening of disease symptoms:

‘Yes, it sometimes happens that for about two weeks I have not taken my drugs. At other times I can go for about one month without my medication because there is no money and it's within those times that the sugar level rises when I go for checkup’ [IDI, female 57 years].

### 3.2 Therapy‐related dimension

The therapy‐related dimension pertains to the difficulties patients face with regard to treatment benefits and side‐effects. Perceived/anticipated benefits of therapy, and actual or perceived side‐effects are important sub‐themes.

#### 3.2.1 Perceived/anticipated benefit of therapy

Some patients indicated that their medications were not providing the desired results – leading them to resort to the use of herbal medication. Patients interviewed had varied perceptions about the benefit of their treatment regimen. To some, any treatment regimen that does not bring instantaneous relief is deemed inefficacious or ineffective. Thus, most sought instant healing of their conditions through herbal therapies (which are perceived to provide relief at a desired rate). Following are illustrative quotes:

‘I took the drugs for some time and stopped and went back to the herbal medicine. I drank about 10 big bottles of the medicine so I realized that I had some form of relief in my bones. I couldn't lift up my hands and legs at first but as time went on I was able to lift my hands and legs and do everything on my own, so the Herbalist told me it's now okay so I should stop taking the medicine so till now I don't take any medicine. What the herbal medicine can do for me in a week, the orthodox does in a year’. [FGD, female, in her 50s].

#### 3.2.2 Actual or perceived side‐effects

Quite a number of patients reported that the side‐effects they experienced while on their medication negatively impacted their motivation to take their medication (Table 1). The quote below is an example of how patients were experiencing headaches and wanting to either stop taking the medication or moving to another one that does not have the side‐effects:

‘The doctor told me that when I take the drug I will experience a slight headache while taking it. Later he asked me to stop taking it because when I take the medicine my head aches and I feel like dying so he asked me to stop’. [IDI, male, 63 years].

**TABLE 1:** Motivations for use of alternative therapies and contributors to non‐adherence to orthodox treatment in patients with established ACVD

Dimensions/themes	Sub theme	Examples of evidence
Social and economic	Transportation and medication costs	*‘I started taking the orthodox medicines after going to the hospital, then my brother's brother in‐law visited one day and asked of my condition, after I had told him about my condition he recommended herbal medicine for me and offered to start buying it for me, if it works then I can start buying it on my own. When I started taking it I could taste garlic and other spices in the herbal solution. I even drank it whenever I was thirsty. After taking it for five days I started having tummy upsets and when I informed him he told me it was the way the drug works, it will wash my system so I continued till I had taken ten bottles. Before then I could not raise my legs and my arm but after taking the tenth bottle I could raise my arm and leg and could walk better, but I stopped after the tenth bottle’*. [IDI, female 60 years]. *‘For the hypertension drugs sometimes I don't get it from the pharmacy at the police hospital. Sometimes I am given a package of the medication for 30 days so when the 30 days elapses then they may give me for two months or I may go and buy it. Sometimes they do tell me if it is finished I can come around and be given a new package but considering the transportation and the process I have to go through to get a folder I don't go’*. [IDI, Female 68 years] *‘………and if by then I don't have money then I will skip a day or two and if I have money I will go and buy it’*. [IDI, Male 59 years]
	Unemployment	*‘‘…okay to me a brother of mine told me that this thing it doesn't happen on people by heart but maybe somebody has done it to you so when you go in spirit they can tell you the cause of your sickness and that's what I am expecting, I’m very, very serious and its true too’’* [IDI, Male, 73 years] *‘When people visit me some give me money, so I put them together and then use that to buy my drugs because now I don't work even the lady am with is not working so I depend on gifts from people to eat and buy my drugs’*. [IDI, female 74 years]
Therapy‐related	Availability of effective treatment	*‘I took the drugs for some time and stopped and went back to the herbal medicine. I drank about 10 big bottles of the medicine so I realized that I had some form of relief in my bones. I couldn't lift up my hands and legs at first but as time went on I was able to lift my hands and legs and do everything on my own, so the Herbalist told me it's now okay so I should stop taking the medicine so till now I don't take any medicine. What herbal medicine does for me in week, the orthodox does in a year’* [FGD, Female in her 50s] *‘Others even ask me how my condition got better, so I have even bought (herbal medication) for like 5 people, all I have to do is to call the man and he will bring it’*. [IDI, female in her 50s] *‘O yes I will go back to herbal. Just to cure’*. [IDI, male 73 years]
	Actual or perceived side‐effects	*‘I was not in agreement with what he proposed. Even though he is a doctor, I wasn't in agreement with him. That medicine was good. He asked me to stop so he will give me a different medicine. The new one he gave me when I took it, I started having headache so I stopped taking it. It even led me to develop stroke. One day whiles lying on bed I experienced this severe headache’*. [IDI, Male in his 60s] *‘I take all of them once a day, there is one I had to take twice but I take it once because of the side effects even though the doctor said twice a day I also take herbs because of the diabetes’* [IDI, male in his 60s] *‘Yes some of the drugs when I take them I begin to feel dizzy for some time and then it makes me sleep and also makes me feel kind of weak’* [IDI, female in her 50s]
Patient‐related	Resources	*‘Yes, at times when I’m busy doing some other chores, I do forget. Sometimes when I’m supposed to take the medication in the morning but I haven't eating early I do forget to take...’* [IDI, female, 67 years] *‘It's only the financial that is only the challenge. At times you will need to do some test but when you check and the money is not going then it means you have to find a way of explaining to the doctor’* [IDI, female in her 60s] *‘Yes it happens sometimes for about two weeks and I have not taken my drugs other times too one month because there is no money and it's within those times that the sugar level rises when I go for checkup’* [IDI, female 57 years]
Health‐care system	Provider communication skills/ provider ‐patient relationship Health insurance	*‘The truth is with the herbal doctors, I was attending the herbal clinic at Dodowa and the way the doctor will treat you and have time to discuss with you it's so nice. He will take his time with you and ask you questions and explained to you all that you need to know. He will tell you want you should eat. He even have a board with drawings of fruit on it telling you what to eat and what not to eat. That place he will tell you everything’* [FGD, Female 62 years] *‘Yes here at police hospital if you carry your health insurance card along they won't give you the drugs so I have to go and buy it outside’* [IDI, female 61 years]
Condition‐related	Chronic condition Perceived importance of treatment	*‘‘I was told when you have stroke it can be treated forever you only keep taking your drugs and even if it will go it will take a very long time if I stop the drugs then I start feeling the pains when I start then it reduces, so if you get hypertension the treatment is not permanent’’*[FGD, Male, 47 years] *‘I think it is all important (medication) because that's her profession. I’m a lay man and I don't know anything about medicine. I think it's the best drugs that she has been prescribing for me’*. [IDI, Male in his 50s] *‘So has there been an occasion where the doctor told you to take a particular amount of dosage in the morning and afternoon but you decided that the dosage wasn't good for you so you altered the dosage by yourself?’* [Interviewer] ‘*I don't do that because I want to be healthy and be able to do things by myself’*. [FGD, Male in his 60s]

### 3.3 Social and economic dimension

This dimension embodies such factors as family dysfunction (lack of family support) and cultural and lay beliefs about illness and treatment etc The dimension also connects with a patient's acceptability and ability to seek alternative forms of treatment for their condition. We provide illustrative quotes pertaining to the sub‐themes of ‘family or social support/network’, and ‘cultural and lay beliefs about illness and treatment’.

#### 3.3.1 Family or social support or social network

Family and friends were found to be successful at introducing patients to herbal medication. The quotes following illustrate the contribution of family or peers to non‐adherence to orthodox therapies:

‘My uncle is a herbalist and he knows much about the herbs so after he saw the scan results, he told them that I had stroke of the head. I was treated for 5 days with the herbal medicines and truly on the 4th day, I was able to move from one point to the other. I went and took my bath and returned to the room. This herbal treatment continued till I started walking again’. [IDI, female, 37 years].

The data also revealed instances where family provided a support structure and facilitated adherence to medication. Below are quotes to support this:

‘My husband does that a lot when I finish eating. He will ask me to go for my drugs’. [IDI, female, 60 years].

‘With my drugs, my children also help me. They ask me if I have taken my drugs. Then I tell them, yes I have’. [IDI, female, 63 years].

#### 3.3.2 Cultural and lay beliefs about illness and treatment

In Ghana, socio‐cultural factors underpin health‐seeking behaviour and dictate the choice and utilization of treatment options for patients with chronic diseases. Culturally informed lay illness models strongly inform individual's attitudes. These can be so deeply held at an individual level that people may remain unaware of them – leaving them assumed shared knowledge and unquestioned. Patients with chronic diseases will visit the hospitals and take‐home orthodox medicine but perceptions they may have on the aetiology and necessary treatment for the condition may preclude them from adhering to orthodox medication. Cultural beliefs surround illnesses that do not resolve even after treatment from a medical doctor or a herbalist. Some illnesses are perceived to be spiritual and require that treatment is sought from faith healers. Some relatives of patients were of the view that their (chronic) condition had been brought upon them by spiritual forces and as such recourse was to be taken against those forces – as illustrated below.

‘Okay, a brother of mine told me that a condition like mine doesn't happen to people without spiritual undertones; maybe somebody has done it to you so when you go to the spirit they can tell you the cause of your sickness and that's what I am expecting’. [IDI, male, 73 years].

There was an instance where patients used orthodox medicine to treat their NCDs and then herbal medication to treat other conditions.

‘Before my present condition, there was a doctor by the Dodowa Road. When I consulted him, I took along all my medication with me. The place was a herbal hospital. When I went there, he asked me to continue taking my BP medications but not some other capsules given to me at the hospital. He said he will give me some other medicine that I will take in place of that. I even stopped going there almost about a year now but after taking the medication he has given to me I was okay. So, it was because of my stomach issues that was why I went there but not because of the heart condition. After attending to me he told me I’m suffering from a heart condition so I should continue taking my medications’. [IDI, female, 68 years].

#### 3.3.3 Faith‐based healing

Of note, our study participants acknowledge that both allopathic and herbal remedies have their limits. Some patients perceived their disease condition to be beyond either medical or herbal therapy. They therefore sought faith‐based healing (prayers) which they regarded as superior to medical or herbal therapy. They note:

‘‘Life is like that. When you get sick and helpless, you have disgraced yourself. God being so good, I didn't come in a wheelchair. I have packed it somewhere in the garage. The Holy Spirit has helped me. He has enabled me to stand because when I lie down, what I hear is what I get up, get up.’ [IDI, female, 63 years].

### 3.4 Health system‐related dimension

The most prominent health system factor observed to affect medication adherence was relationship between the health‐care provider and the patient. It was observed that patients will have liked their doctors to provide information about their condition. Others found that herbalist made time to explain their disease condition and answer their queries. The below dialogue illustrates.

‘Does he (doctor) spend time to have discussion with you concerning your condition so that you will understand your condition very well and also explain all that you should do and not do to live healthy?’ [Interviewer].

‘No, even when you go to the hospital there are lot of people there so he has to be fast

with you so that other people will also have the turn to see him. Because the people are

many, the discussion will waste someone's time’. [IDI, female, 67 years].

Others found the herbal clinics were more receptive to their needs and that herbal doctors took time and answered their queries.

‘The truth is that, with the herbal doctors, I was attending the herbal clinic at Dodowa and the way the doctor will treat you and have time to discuss with you it's so nice. He will take his time with you and ask you questions and explain to you all that you need to know. He will tell you want you should eat. He even had a board with drawings of fruit on it, telling you what to eat and what not to eat. He will tell you everything’. [FGD, female, 62 years].

### 3.5 Condition‐related dimension

Condition‐related factors represent the particular illness‐related demands faced by the patient such as the chronic nature of the condition and the severity of symptoms of the condition. Our data revealed that the chronic nature of the condition and the severity of symptoms acted as facilitators of medication adherence. Below are quotes to support this:

‘‘I was told when you have stroke it can be treated forever you only keep taking your drugs and even if it will go, it will take a very long time. if I stop the drugs then I start feeling the pains when I start then it reduces, so if you get hypertension the treatment is not permanent.’’ [FGD, male, 47 years].

‘It is helping. Example for the aspirin if I wake up and I forgot to take it then I start to feel dizzy but when I remember and I go and take it then I feel okay’. [IDI, Female, 67 years].

## 4 DISCUSSION

Non‐adherence to medication among patients with chronic ailments is a widespread practice in Ghana.^25^, ^47^, ^50^ This practice greatly affects health outcomes of such patients. In order to improve medication taking behaviour, it is important to understand the reasons for non‐adherence. This paper presents data on our exploration of the causes of poor adherence to medication intake in patients with established atherosclerotic cardiovascular disease (ACVD). Organized according to the WHO five interacting dimensions of adherence, the current study shows that non‐adherence is precipitated by a multi‐factorial interplay of determinants – patient‐related factors including the quest for curative measures, family support and social network, the health‐care system and the treatment‐related factors.

The WHO framework identifies the fact that cultural and lay beliefs of patients about illness are embedded in socio‐cultural factors which underpin health‐seeking behaviour and influence the choice and utilization of treatment options of patients of chronic diseases.^22^ Perception about the duration of the disease and aetiology of the disease all affect health‐seeking behaviour. In Africa, illness perception goes beyond the Western biomedical approach. It is much more holistic involving not just the body but the mind and sometimes the supernatural.^51^ Healing practices also serve as a representation of cultural beliefs, which influence health behaviours and serve as a framework for interpreting disease conditions.^52^ As stated in the previous paragraph, the current study identified that patients considered certain sicknesses as requiring spiritual attention for healing to take place.

Some patients with ACVD perceived herbal medication as possessing the ability to cure their condition and mostly preferred it over orthodox medication in some instances even in the face of adverse effects. As observed in the current study, *episodes of diarrhoea experienced by a participant, which she attributed to the use of a herbal concoction, did not deter her from using it. Rationalization from her brother‐in‐law that such episodes of diarrhoea demonstrate the efficacy of the concoction, kept her going till the 10th bottle*. In Ghana, this phenomenon has been noted by local sociologists, to have cultural underpinnings where medications deemed efficacious, ‘have to first knock you off your feet and then bring you up’ *(Professor Kodjo Senah, Personal Communications)*. This goes to show the strong cultural attachments to the use of such medication. Our findings compare with those of a study carried out in Ghana where hypertension was considered as not being a ‘hospital disease’ but a disease that required the use of herbal medicine to cure the condition.^53^ Elsewhere, traditional faith healers are sought for cures for diseases such as diabetes,^54^ hypertension^55^ and other complications of cardiovascular diseases such as stroke.^56^

In Ghana, diseases which persist after the patient has received treatment from a medical doctor or herbalist are described as spiritual.^47^, ^57^ Such diseases are thought to require divine intervention resulting in non‐adherence to orthodox therapies. The current study revealed that some patients preferred faith‐based healing because of the promise of a cure to their health condition and the belief that the sickness had spiritual causations beyond medical treatment. The problem with faith‐based healing is the conflict with medical care. It results in the belief that medication is unnecessary because ill health is an act of God and is uncontrollable except through divine intervention. This was also observed in the current study where some patients were observed to have discontinued medication in favour of prayers.

The current study identified inadequate communication between patient and health‐care professionals as a contributor to medication non‐adherence. It was revealed that the sheer numbers of patients visiting the hospitals for medical care precluded health professionals from educating patients on their medication usage as well as side‐effects and their health condition. In a similar study conducted to determine patients’ perceptions of medication adherence, it was identified that a lack of education and patients’ fears of medication side‐effects had the potential of adversely affecting medication adherence.^58^

This study also observed that, among ACVD patients, the use of herbal alternatives was not a primary choice. Most of them sought treatment in hospitals where they were given medication. The use of herbal alternatives came about when they could not obtain instant relief from the symptoms of their condition while taking the orthodox medication. This finding compares favourably with that found in similar studies carried out in Ghana.^25^, ^50^

Perceived medication inefficacy was another barrier to medication adherence. This finding was observed in another qualitative study which was carried out on TB patients in Ghana where the patients were observed to be impatient to take their medications as prescribed.^59^ The current study showed that some patients sought to augment the orthodox medication with herbal medication. Similar studies have also shown that doubt of treatment efficacy, distrust for medication and adverse effects were major causes of non‐adherence to medication.^60^, ^61^ This was also observed in the current study.

Our results are consistent with other studies reporting that social support directly affects adherence to orthodox medication. A study on healer shopping for diabetes in Ghana found that despite a preference for biomedical medication, the socio‐economic impacts of diabetes resulted in promoting a cure seeking behaviour in patients.^50^ The results of a similar study carried out in rural Ghana showed that socio‐cultural and economic factors hinder social support and the patient's own social relations with family and or community members determine to an extent the social support he or she receives for their health condition.^53^

### 4.1 Medical pluralism and adherence to orthodox therapies

As outlined earlier patients in Ghana have the opportunity to shop for healing from various systems/care providers – the allopathic health services delivery system, indigenous/traditional care delivery systems and faith‐based providers. Referred to as medical pluralism^62^ or medical diversity,^63^ the use of multiple medical systems to address illness and wellness has been discussed, rather controversially. The strongest criticism has come from critical medical anthropology whose proponents stressed patterns of hierarchy and the dominance of biomedicine in the modern world, calling into question the notion of ‘pluralism’ itself.^64^ Aside that, there is an ongoing debate about the contribution of indigenous/traditional healers, in chronic disease care in Africa.^65^ In Ghana, the practice of allopathy is often framed to compete with alternatives such as traditional/indigenous and faith‐based remedies.^66^ Awah and Phillimore^67^ explored the tension between clinic‐based demands for patients' ‘compliance’ with treatment guidelines, including repeated strictures against resorting to ‘traditional’ medicine, and patients' own willingness to alternate between biomedicine and indigenous practitioners. Their work shows how traditional medicine can be something to work with and how it can improve situations, rather than regarding it as an ineffective opposition.

Critics of indigenous medicine point out its implications for service uptake and adherence to allopathic care. In the current study, potential barrier to uptake and adherence to ACVDs medications relate to the belief that traditional medicines have curative potential and spontaneity of action. We concur with de‐Graft‐Aikins^66^ on this subject. de‐Graft‐Aikins reported that the patients she engaged unfortunately set out to seek a ‘cure’ for an ‘incurable’ disease like diabetes. Similar claims are rife with other NCDs and in other African settings.^65^ On the basis of these, we recommend meaningful engagement of stakeholders of this pluralistic health‐care system (allopathic care providers, indigenous/alternative medicine care providers, policy makers, etc) so as to facilitate a nuanced understanding of the issues.

### 4.2 Limitations of study

The study was conducted in one hospital in Accra the capital of Ghana and this could limit generalization of the findings due to the fact that medication adherence is a complex issue and could vary from setting to setting. Second, although the qualitative research assistants were trained to handle courtesy bias or socially desirable responses on the part of all respondents, we are not able to wholly rule it out. Despite these limitations, this paper sheds light on important aspects of adherence to orthodox therapy in patients with ACVD in a cosmopolitan Ghanaian setting. These have the potential to impact practice, policy and further research.

### 4.3 Implications for practice, policy and future research

Culture shapes health behaviours and serves as the lens by which experiences are interpreted and perceived.^68^ Understanding the cultural framework by which disease is interpreted is important in improving adherence to orthodox medication in ACVD patients. The WHO in the year 2002 identified the integral part traditional medicine plays in the health of Africans.^69^ The Ghana Herbal Pharmacopoeia (GHP) for example reported that due to various challenges with the orthodox health‐care delivery system, at least 70% of Ghanaians depend on alternative medicine for their health needs.^40^ Integrating traditional and orthodox medicine therefore expands the reach and enhances the result of community health care.^70^ In Ghana, attempts to integrate traditional and orthodox medicine have proved futile for reasons such as lack of education on the part of traditional medicine practitioners and the challenges with enforcing rules and ethics.^71^, ^72^ There are however opportunities for integration which can be considered. These could include enhancing medical doctors' sensitivity towards traditional healing and encouraging traditional healers to develop some collaborative work with medical doctors. There are various ways in which traditional healers can complement medical doctors, for example: acting as referral points, sending patients for biomedical treatment; discouraging traditional healers from any practices that may potentially harm the patients; and encouraging and monitoring adherence to the treatment regimen recommended by medical doctors.^69^

Health provider–patient communication is another area which requires attention. The study revealed that the high patient burden on medical practitioners precluded effective communication with ACVD patients. Studies carried out to assess the feasibility of training of nurses in task shifting strategies for the management of chronic diseases like hypertension are a step in the right direction and their results should be implemented speedily.^73^

To conclude, this study identified widespread use of herbal therapy (most of the time concomitantly with orthodox medication, and faith‐based healing) by patients with established ACVDs. This is done in an attempt to treat and ‘cure’ their condition. As argued by Laar et al,^74^ until all stakeholders the pluralistic Ghanaian health‐care system are made to understand that there is as yet no cure for such conditions, this phenomenon of seeking cures for ‘incurable’ health conditions will continue. These findings have important implications for orthodox medication adherence as far as the concomitant use of herbal preparations is concerned.

## 5 PATIENT OR PUBLIC CONTRIBUTION

Clinicians and patients contributed to a co‐development of a text message intervention to support patients with ACVD in Ghana adhere to treatment.

## CONFLICT OF INTEREST

All authors declare no conflict of interest.

## AUTHOR CONTRIBUTIONS

PP conceived of the project and led the design of the study. AL, PL, HL‐Q, and YF contributed to the design of the study. AL, EAA, GSA, PN, and FB supervised the implementation of the field research. YF, EAA, GSA, AL, and HL‐Q contributed to data management and analysis. AL and MA drafted the manuscript, with significant inputs from all co‐authors. All authors reviewed and approved the final version of the manuscript.

## FUNDING INFORMATION

The TXT2Heart pilot study was funded by the Joint‐Global Health Trials scheme from MRC/UKaid/Wellcome Trust from UK for pilot work in Colombia, Ghana and India.

## Data Availability

Data are available on request from the authors: The interview transcripts that support the findings of this study are available from the corresponding author upon request.
